# Everybody's Page

**Published:** 1891-07-18

**Authors:** 


### Everybody's Page.

The Irishman's idea of liberty is to do just what he pleases, when he pleases and where he pleases, and make everyone else do the same.

A good illustration of the expansion of the world's trade during the past thirty years is afforded by the production of petroleum in the United States. In 1859, 84,000 gallons were produced in the Pennsylvania and New York oil fields, and in 1890, 689,029,966 gallons were exported from the various states which now produce the oil.

Mr. G. J. Symons, F.R.S., has found a new elixir of life. It is within the reach of the humblest of Her Majesty's subjects, and has the merit of simplicity. It consists in the study of meteorology, which Mr. Symons has observed has a remarkable effect in prolonging longevity. The climate of this country is an excellent field for the employment of this elixir.

The ideas on sanitation of the inhabitants of Colorado are simple, if crude. The neighbourhood of West Denver having been for some time unhealthy, an examination was made of some cellars occupied by the Chinese colony. The examination proved unpleasant to all parties concerned, more especially to one particular Chinamen, and he would have been hanged by the enraged populace, whose olfactory nerves had been so upset, but for the timely interference of the police. A drastic, but hardly effective or scientific, method of improving the sanitary arrangements of the City!

According to the *Medical Record* the practice is far too prevalentin the hospitals of the United States of labelling as "hysteria" cases that exhibit a little too much moral perversity or emotional excitement. A good deal of injustice and neglect sometimes result from this habit, which has its counterpart occasionally in this country when Constable Z apprehends as drunk or incapable some unfortunate man or woman who is suffering from an epileptic fit. Some time ago it was stated in the New York papers that a woman had committed suicide by drinking a quantity of belladonna liniment, and that the ambulance surgeon mistook the case at first for hysteria.

It is stated that a national Italian Pharmacopœia is shortly to be published. Hitherto the formularies in use in Italy have consisted for the most part of translations from French formularies.

A new dispensing balance has been brought out by Mr. Nithack, a chemist of Obernigk, in the government district of Breslau, which is said to be as simple and durable as it is accurate and moderate in price. It is being manufactured by Winkler and Jenke, of Berlin.

A number of experiments with artificial digestive fluids have led Dr. Hugounenq to the conclusion that all wines without exception interfere with the action of pepsin, those which are most highly charged with colouring matter, alcohol, and cream of tartar, being most hurtful, and that the acidity of new wines is incapable of aiding the action of pepsin.

The Chicago Pasteur Institute for the preventive treatment of hydrophobia is to be congratulated on its success. During the first ten months of its existence 55 persons have received treatment, and all are now enjoying good health. The exercise of ordinary common, or rather uncommon, sense has no doubt contributed to this result. It has been the invariable practice of the Institution never to submit persons to treatment unless they produce reasonable evidence to demonstrate the madness of the animals which have bitten them.

Count Tolstoi's essay on narcotics and stimulants has called forth much discussion and many varied opinions in Paris. M. Alphonse Daudet thinks that the Count has, as usual, seen everything larger than life, and has been carried away by his feelings in his crusade upon the effect of tobacco on the intellectual and moral faculties. Abuse of tobacco and alcohol, like abuse of all things, is no doubt folly. M. Daudet thinks that after dinner nothing is so good as a pipe, taken with one or two little glasses of excellent brandy. We think the pipe without the brandy is still better, and less injurious. M. Zola does not drink wine—some people may be astonished to hear this—but he does not suppose that this makes him wise. The Count will not find many people to agree with his view that the habit of intoxication is indulged in from an instinctive desire to stifle the moral conscience.

